# Paraneoplastic Pemphigus Unveiling Occult Lung Adenocarcinoma: A Rituximab‐Responsive Case With Diagnostic and Therapeutic Implications

**DOI:** 10.1002/ccr3.71386

**Published:** 2025-10-30

**Authors:** Yongfeng Li, Haochuan Ma, Xinsheng Chen, Yihong Liu, Yu Zhang, Junzhe Li, Hanliang Zhang, Liting Zhang, Shuyun Xiong, Jicai Chen

**Affiliations:** ^1^ Department of Thoracic Surgery The Second Affiliated Hospital of Guangzhou University of Chinese Medicine, Guangdong Provincial Hospital of Chinese Medicine Guangzhou Guangdong China; ^2^ Department of Oncology The Second Affiliated Hospital of Guangzhou University of Chinese Medicine, Guangdong Provincial Hospital of Chinese Medicine Guangzhou Guangdong China; ^3^ Department of Dermatology The Second Affiliated Hospital of Guangzhou University of Chinese Medicine, Guangdong Provincial Hospital of Chinese Medicine Guangzhou Guangdong China; ^4^ Department of Pathology The Second Affiliated Hospital of Guangzhou University of Chinese Medicine, Guangdong Provincial Hospital of Chinese Medicine Guangzhou Guangdong China; ^5^ Department of Imaging The Second Affiliated Hospital of Guangzhou University of Chinese Medicine, Guangdong Provincial Hospital of Chinese Medicine Guangzhou Guangdong China; ^6^ Department of Anesthesiology The Second Affiliated Hospital of Guangzhou University of Chinese Medicine, Guangdong Provincial Hospital of Chinese Medicine Guangzhou Guangdong China

**Keywords:** case report, EGFR‐mutated, lung adenocarcinoma, paraneoplastic pemphigus, rituximab

## Abstract

Paraneoplastic pemphigus (PNP) is a rare autoimmune disorder typically associated with hematologic or squamous cell malignancies. Its association with lung adenocarcinoma (LUAD) is exceptionally uncommon, with only sporadic cases reported. This report highlights PNP as a sentinel manifestation of occult LUAD, emphasizing diagnostic challenges and therapeutic implications in this underrecognized association. A 73‐year‐old Asian woman presented with refractory mucocutaneous erosions, confirmed as PNP through histopathology (intraepidermal acantholysis), direct immunofluorescence (IgG/C3 deposits), and elevated anti‐desmoglein‐3 antibodies (172 U/mL). Persistent symptoms prompted malignancy screening, which revealed stage IA EGFR‐mutated LUAD. Initial immunosuppression (corticosteroids, intravenous immunoglobulin) failed to control disease. Tumor resection induced transient remission, but postoperative recurrence required rituximab therapy, achieving sustained clinical and serologic improvement (anti‐Dsg3 decline to 60 U/mL). This case expands the known malignancy spectrum of PNP to include LUAD and underscores the necessity of rigorous tumor screening even in atypical presentations. The temporal correlation between tumor resection, rituximab response, and autoantibody decline supports synergistic neoplastic/immune mechanisms. Persistent B‐cell activity post‐resection suggests adjunctive immunomodulation is critical for durable remission. Our findings advocate for (1) multidisciplinary collaboration to address diagnostic pitfalls (e.g., lesion mimicry, delayed tumor detection) and (2) consideration of rituximab in refractory PNP with solid tumors.

## Graphical abstract

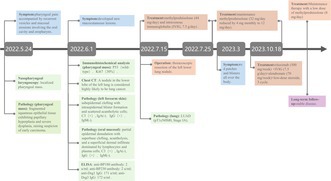

**Summary**

- Paraneoplastic pemphigus, marked by suprabasal clefting and dermoepidermal junction inflammation, may reveal occult lung adenocarcinoma.
- Comprehensive malignancy screening, tumor resection, and rituximab therapy achieve remission, with serologic monitoring guiding treatment.
- Multidisciplinary collaboration is crucial for timely diagnosis and effective management of this rare autoimmune disorder.

## 1 Introduction

Paraneoplastic pemphigus (PNP) is defined as a rare autoimmune disorder characterized by severe mucocutaneous blistering and erosions, almost always associated with an underlying neoplasm [1, 2]. It involves autoantibodies targeting components of the desmosome and hemidesmosome, leading to the disruption of cell–cell adhesion in epithelial tissues [3]. This results in painful lesions, including oral and skin erosions, flaccid blisters, and sometimes lichenoid or erythema multiforme‐like eruptions. The disease can also affect the eyes, lungs, and gastrointestinal tract, with potential complications like bronchiolitis obliterans in severe cases. Clinically, the presentation varies, often starting with severe diffuse blisters in the mouth and lips, and its mimicry of conditions such as lichen planus or erythema multiforme frequently delays diagnosis [4, 5, 6].

The incidence of PNP is notably low, accounting for approximately 3%–5% of all pemphigus cases, with around 500 cases reported globally [7, 8]. It is most frequently linked to lymphoid malignancies like non‐Hodgkin's lymphoma and chronic lymphocytic leukemia, but less commonly with solid tumors [9]. Notably, the association of PNP with lung cancer, especially lung adenocarcinoma (LUAD), is even rarer, with case reports suggesting a stronger association with lung squamous cell carcinoma (LUSC) or other histological types [10, 11, 12, 13]. This case report details a 73‐year‐old woman in whom PNP with persistent skin and mouth lesions led to the discovery of stage IA EGFR‐mutated pulmonary adenocarcinoma during diagnostic evaluation. This finding is unexpected, because lung adenocarcinoma is less commonly associated with PNP compared to other cancers, thereby making this a significant observation for future research and diagnostic strategies.

## 2 Case History/Examination

**FIGURE 1:**
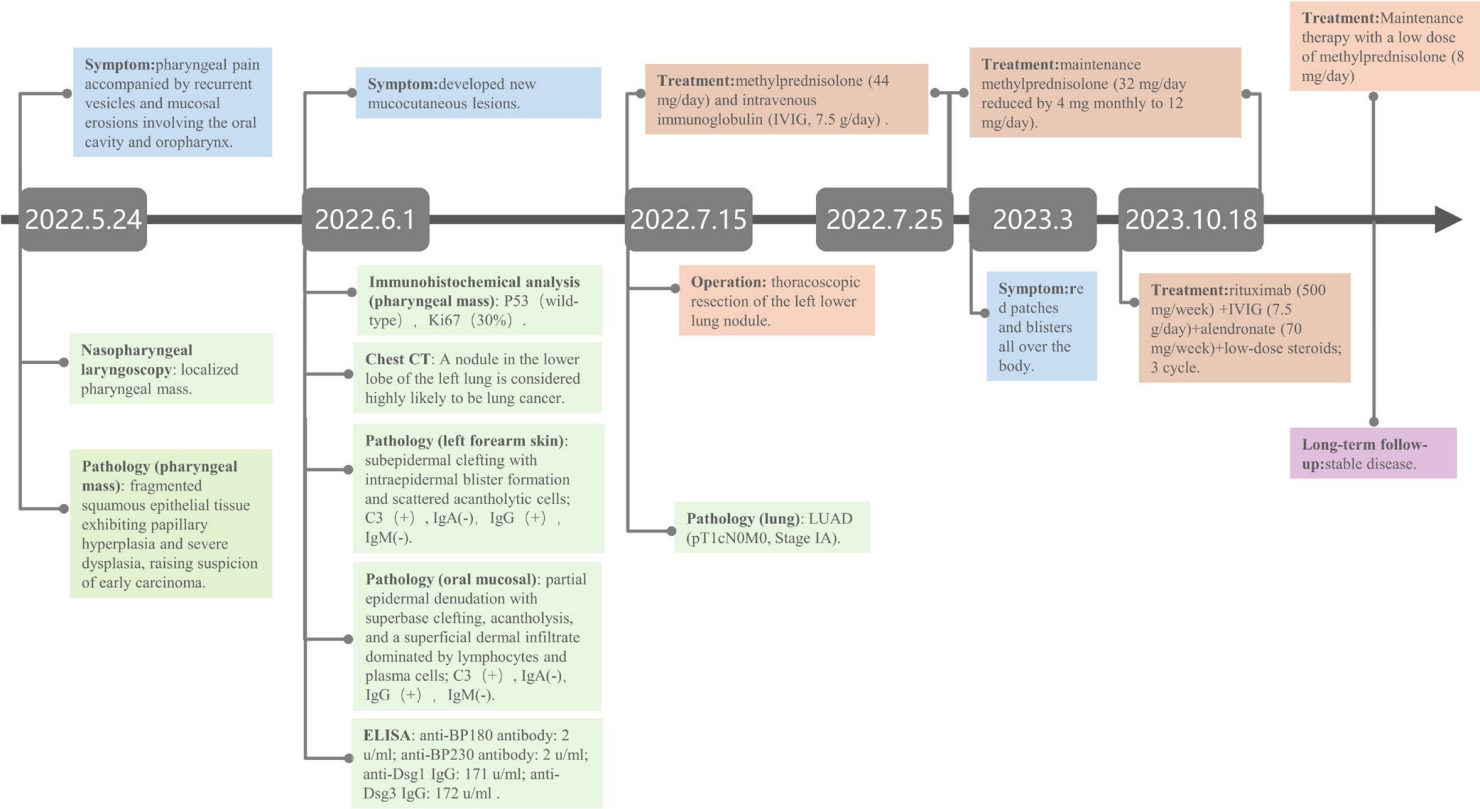
Overview time line of the patient's clinical history. IVIG, intravenous immunoglobulin; LUAD, lung adenocarcinoma.

**FIGURE 2:**
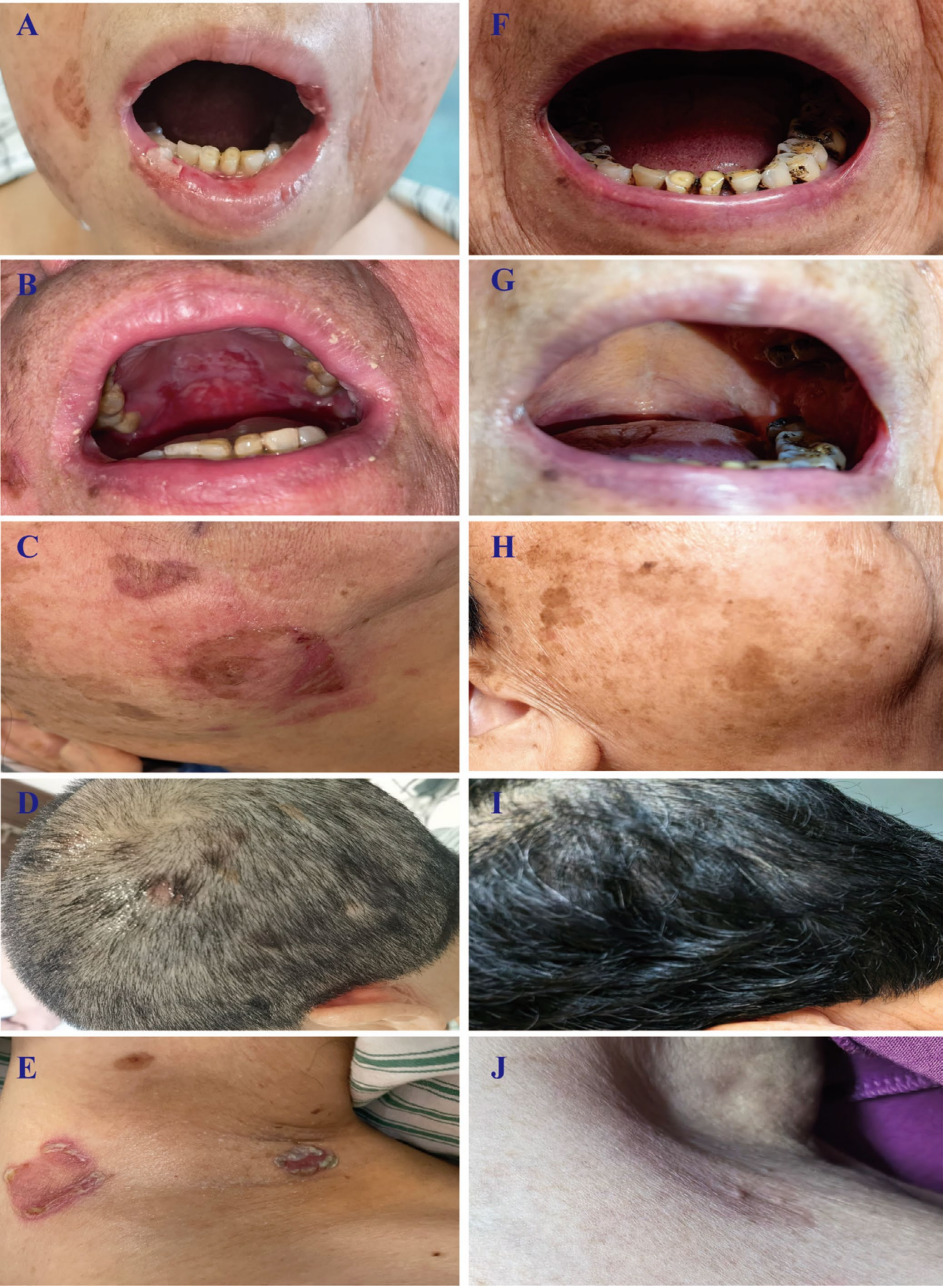
Representative photographic images of cutaneous lesions before and after treatment. (A–E) Initial presentation showing characteristic lesions affecting: (A) vermilion border of the lip, (B) buccal mucosa, (C) malar region, (D) vertex scalp, and (E) axillary fold. (F–J) Corresponding anatomical sites at final follow‐up (34 months post‐treatment): (F) perioral region, (G) oral cavity, (H) facial dermatome, (I) parietal scalp, and (J) axilla. All images were obtained under standardized photographic conditions.

An overview of the patient's clinic history is shown in Figure 1. A 73‐year‐old woman presented to the otolaryngology clinic on May 24, 2022, with a one‐month history of progressive pharyngeal pain accompanied by recurrent vesicles and mucosal erosions involving the oral cavity and oropharynx (Figure 2A,B). Systemic symptoms such as fever, chest tightness, or cough were absent. Initial diagnostic imaging with laryngoscopy identified a localized pharyngeal mass. Histopathological examination of the biopsy specimen revealed fragmented squamous epithelial tissue exhibiting papillary hyperplasia and severe dysplasia, raising suspicion of early carcinoma. However, due to the limited quantity and suboptimal preservation of the biopsy material, a definitive diagnosis could not be established. The pathology report explicitly recommended clinical correlation and repeat sampling if symptoms persisted.

**FIGURE 3:**
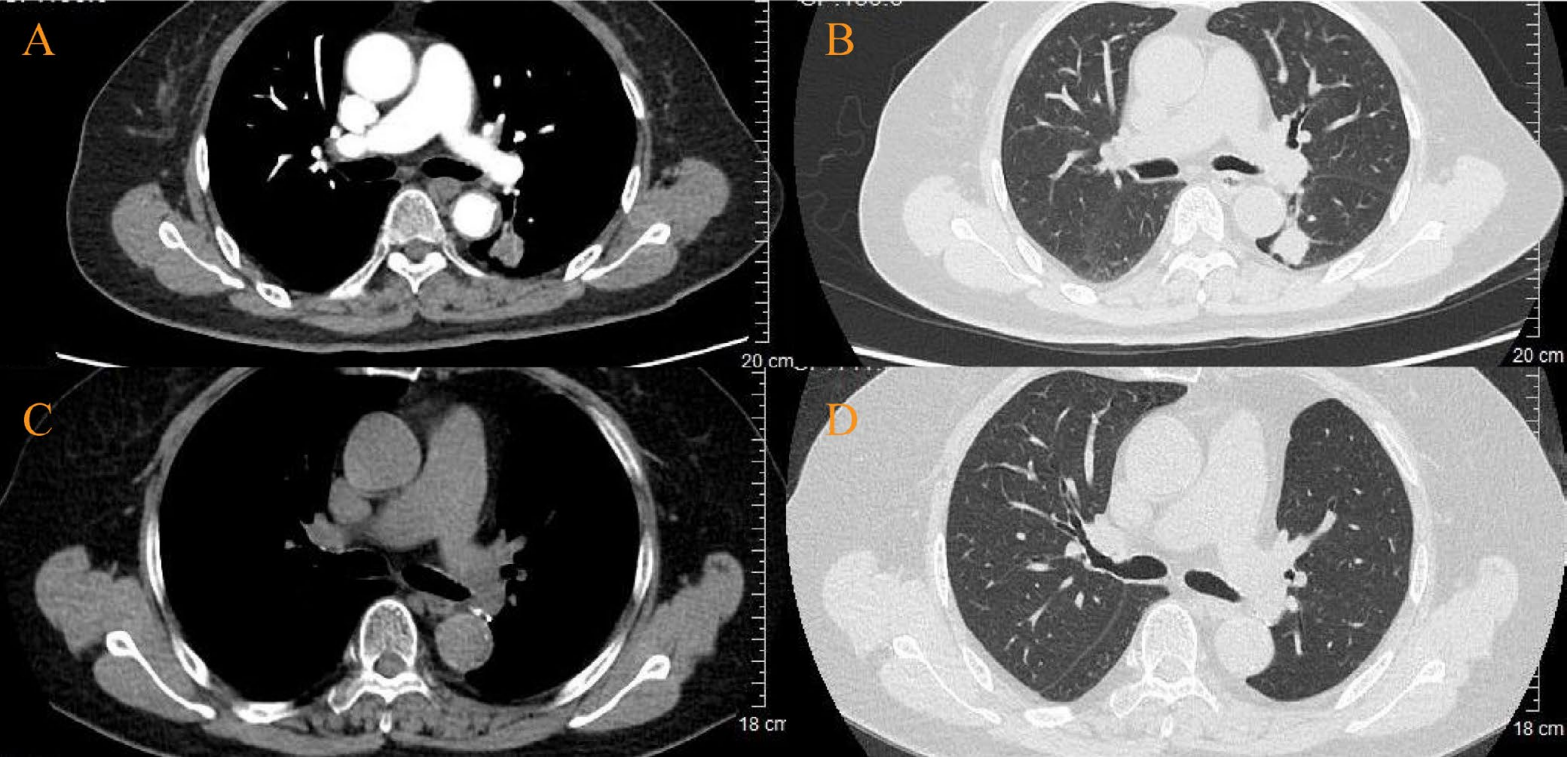
Radiologic evolution of left lower lobe lung tumor pre‐ and post‐surgical resection. (A, B) Baseline contrast‐enhanced axial chest CT at diagnosis. (C, D) Postoperative surveillance CT at 34‐month follow‐up.

**FIGURE 4:**
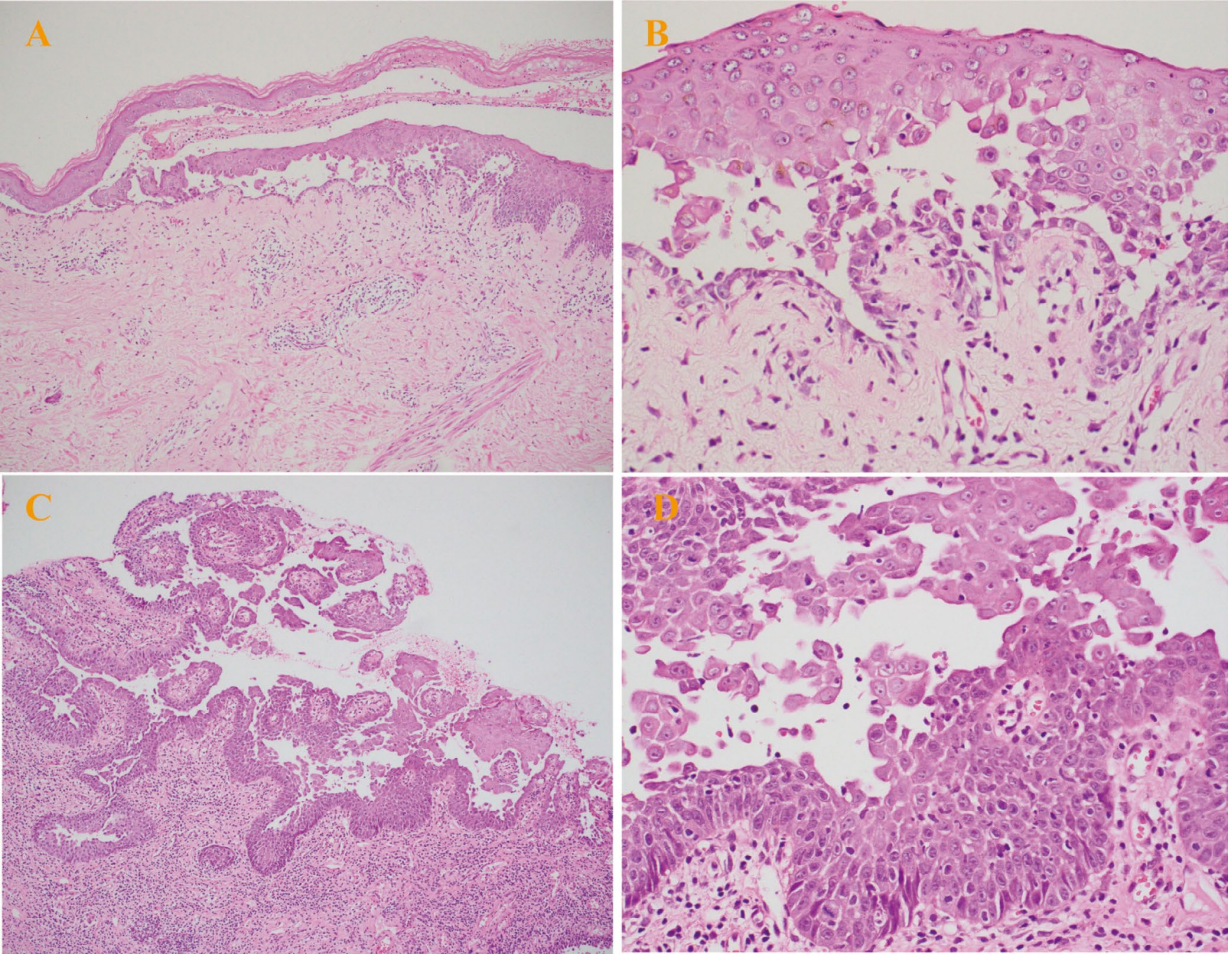
Histopathological features of cutaneous and oral mucosa lesions at initial diagnosis. (A, B) Left volar forearm skin biopsy: (A) 100× H&E staining. (B) 400× H&E staining. (C, D) Oral mucosa biopsy: (C) 100× H&E staining. (D) 400× H&E staining.

The patient's condition deteriorated over the following week, culminating in hospitalization on June 1, 2022, when she developed new mucocutaneous lesions, including painful vulvar erosions and widespread erythematous plaques with flaccid blisters and erosions on the trunk (Figure 2C–E). To clarify the diagnosis, a systematic diagnostic evaluation was conducted: (1) Immunohistochemical analysis of the pharyngeal mass demonstrated wild‐type *P53* protein expression, ruling out *TP53*‐associated carcinogenesis, and a *Ki‐67* proliferation index of 30%, consistent with moderate cellular activity. (2) Subsequent contrast‐enhanced chest CT disclosed a solitary 2.0 × 1.7 cm nodule in the left lower lung lobe, characterized by spiculated margins and heterogeneous enhancement, radiologically compatible with primary lung malignancy (Figure 3A,B). (3) Histopathological examination of a left forearm skin biopsy revealed suprabasal clefting with intraepidermal blister formation and scattered acantholytic cells, diagnostically aligned with pemphigus‐like injury. Notably, additional features included focal vacuolar degeneration of basal keratinocytes and a dense perivascular lymphocytic infiltrate at the dermoepidermal junction. Direct immunofluorescence (DIF) demonstrated linear IgG and granular C3 deposits localized to the lower two‐thirds of the epidermis, confirming an immune‐mediated blistering disorder (Figure 4A,B). (4) Parallel evaluation of oral mucosal biopsy showed partial epidermal denudation with suprabasal clefting, acantholysis, and a superficial dermal infiltrate dominated by lymphocytes and plasma cells. DIF of oral tissue revealed IgG and C3 deposits encircling isolated acantholytic cells, further supporting autoimmune pathology (Figure 4C,D). (5) At initial malignancy work‐up (June 2022), serum tumor markers were as follows: SCC 4.6 ng/mL (normal < 2.5), CYFRA 21–1 2.9 ng/mL (normal < 3.3), NSE 12.1 ng/mL (normal < 16.3), and CEA 18.2 ng/mL (normal < 5). (6) Serologic profiling detected elevated anti‐desmoglein antibodies (anti‐Dsg1 IgG: 171 U/mL [normal < 20]; anti‐Dsg3 IgG: 172 U/mL [normal < 20]), while anti‐BP180 and anti‐BP230 antibodies were within normal limits. Plakin‐specific antibody assays were not performed due to limited archival tissue availability and laboratory constraints. However, despite anti‐Dsg1/3 positivity, the combination of (a) multi‐site refractory mucosal involvement (oral, vulvar, truncal), (b) histopathology demonstrating suprabasal clefting with interface dermatitis and dense lymphoplasmacytic infiltration, (c) DIF showing linear/granular IgG and C3 deposits along the lower two‐thirds of the epidermis, and (d) temporal association with IA‐stage EGFR‐mutant lung adenocarcinoma, supports a PNP diagnosis over pemphigus vulgaris (PV). As previously reported, a subset of PNP cases may exhibit high‐titer anti‐Dsg1/3 even without plakin assays [6, 14].

## 3 Differential Diagnosis, Investigations and Treatment

Initial therapy with high‐dose methylprednisolone (44 mg/day, equivalent to 0.73 mg/kg/day for a 60 kg patient) and intravenous immunoglobulin (IVIG, 7.5 g/day × 5 days, equivalent to 0.125 g/kg/day × 5 days for a 60 kg patient) was initiated. The methylprednisolone dose was titrated to 44 mg/day based on the patient's partial clinical response to an initial 48 mg/day dose, balancing efficacy with minimization of side effects due to her advanced age and comorbidities. The IVIG dose, lower than the guideline‐recommended 0.4 g/kg/day × 5 days, was selected due to patient tolerance, cost constraints, and institutional practice. This regimen failed to achieve disease control, prompting surgical intervention.

On July 15, 2022, the patient underwent video‐assisted thoracoscopic resection of the left lower lung nodule. Histopathological analysis confirmed moderately to poorly differentiated LUAD (pT1cN0M0, Stage IA). Molecular profiling identified an EGFR exon 19 deletion (p.E746_A750del). Postoperatively, immunosuppressive therapy with methylprednisolone and IVIG was continued, resulting in gradual resolution of mucocutaneous lesions over 3 weeks. The patient was discharged on July 25, 2022, on a structured steroid taper (32 mg/day reduced by 4 mg monthly to 12 mg/day).

In March 2023–8 months after discharge—the patient experienced disease recurrence, manifesting as left periorbital erythema and vesicles that expanded to involve the ipsilateral face, nasal bridge, umbilicus, and oral mucosa. Despite ongoing maintenance methylprednisolone (12 mg/day), lesions persisted for 7 months. On October 18, 2023, considering the patient's advanced age, comorbidities, and financial concerns, rituximab (RTX) 500 mg was administered once weekly for 3 weeks (instead of 1 g × 2 weeks apart or 375 mg/m^2^ weekly for 4 weeks). Concurrently, IVIG (7.5 g/day × 5 days, equivalent to 0.125 g/kg/day × 5 days for a 60 kg patient) and low‐dose methylprednisolone (12 mg/day) were given. Although international guidelines recommend RTX at 1 g on days 0 and 14 or 375 mg/m^2^ weekly for 4 weeks, our modified regimen achieved rapid and sustained clinical remission by Week 6.

## 4 Conclusion and Results (Outcome and Follow‐Up)

The patient has remained under close surveillance through serial clinical, radiologic, and serologic assessments. High‐resolution CT scans performed biannually showed no evidence of pulmonary recurrence or metastatic disease. Serologic monitoring revealed a progressive decline in anti‐Dsg3 IgG titers from 172 U/mL (preoperative) to 60 U/mL (post‐RTX), directly correlating with sustained clinical remission. As of March 26, 2025, the patient maintains stability on low‐dose methylprednisolone (8 mg/day), with no evidence of cutaneous/mucosal relapse or tumor recurrence (Figures 2F–J and 3C,D).

## 5 Discussion

PNP is a rare and diagnostically challenging autoimmune disorder, often associated with occult malignancies. This case of PNP preceding the diagnosis of stage IA EGFR‐mutated pulmonary adenocarcinoma illustrates the clinical and therapeutic complexities of this condition and provides observations relevant to its management and pathophysiology.

The diagnosis of PNP requires careful integration of clinical, histopathological, and serological findings [15]. In this patient, the presence of mucocutaneous erosions (oral, vulvar, and truncal), histopathological evidence of acantholysis and interface dermatitis, and DIF showing IgG/C3 deposits at the epidermal junction met established diagnostic criteria. The absence of anti‐BP180/230 antibodies, which are typically elevated in bullous pemphigoid, contrasted with elevated anti‐desmoglein 1/3 antibodies (anti‐Dsg1: 171 U/mL; anti‐Dsg3: 172 U/mL), confirming the characteristic autoantibody profile of PNP. However, the initial resemblance of oral lesions to benign conditions such as lichen planus delayed malignancy detection, underscoring the importance of considering PNP in patients with refractory mucosal erosions. This diagnostic delay highlights the necessity of multidisciplinary collaboration: laryngoscopy and subsequent chest CT—prompted by persistent symptoms—were critical in identifying the underlying lung adenocarcinoma.

Therapeutic management of PNP remains challenging. While high‐dose corticosteroids and IVIG are first‐line therapies, mucosal lesions often respond poorly, as exemplified in this case [16]. Surgical resection of the lung adenocarcinoma led to transient improvement in mucocutaneous lesions, suggesting tumor‐derived antigens may contribute to autoimmune activity. However, disease recurrence 8 months post‐resection revealed that residual autoreactive B‐cell clones persist despite tumor removal. The subsequent response to RTX, a B‐cell‐depleting agent, aligns with its efficacy in pemphigus vulgaris, where it reduces pathogenic autoantibody titers. In this patient, anti‐Dsg3 levels declined from 172 U/mL to 60 U/mL post‐RTX, correlating with clinical remission. While this outcome supports the use of RTX in refractory PNP, further studies are needed to clarify its long‐term role in malignancy‐associated cases.

The association of PNP with LUAD is noteworthy, as most reported cases involve squamous cell carcinoma (SCC) [10, 11, 12, 13]. While SCC shares keratinocyte‐derived antigens with epithelial targets of PNP autoantibodies, LUAD's glandular origin suggests alternative mechanisms of immune dysregulation [14, 17]. Notably, the presence of an EGFR exon 19 deletion in this tumor raises questions about whether oncogenic pathways influence antigen exposure or epitope spreading. Although speculative, EGFR‐driven cytoskeletal remodeling might theoretically expose cryptic antigens, potentially triggering cross‐reactivity [18]. This case emphasizes the importance of malignancy screening in PNP, even for tumor types with atypical associations.

Clinically, this case reinforces three considerations: (1) Early malignancy detection is critical, as tumor resection may reduce antigenic triggers of autoimmunity. (2) Persistent mucocutaneous lesions in PNP warrant systemic evaluation for occult neoplasms. (3) Adjuvant immunomodulation (e.g., RTX) may be necessary to address residual autoimmune activity post‐tumor resection. The correlation between anti‐Dsg3 titers and clinical activity in this patient suggests serologic monitoring could aid in assessing treatment response, though larger studies are needed to validate this approach.

## 6 Limitations

(1) Plakin antibody assays via Western blot or rat bladder IIF were not performed due to limited tissue availability. Future studies should include plakin testing for definitive confirmation. (2) The RTX and IVIG regimens used in this retrospective case differ from standard international protocols due to patient‐specific factors and resource constraints. This may affect direct comparisons of efficacy; future prospective studies should explore optimal dosing strategies. (3) As this is a retrospective case from 2022, no HSV‐1/HSV‐2 PCR swabs or lesion viral cultures were performed at that time. Consequently, we cannot entirely exclude the possibility of concomitant viral infection. Future prospective cases should include routine HSV and VZV screening to rule out viral co‐infection.

In conclusion, this case underscores the diagnostic and therapeutic challenges of PNP, particularly when associated with rare malignancies such as LUAD. While surgical resection of the tumor and RTX therapy achieved sustained remission, the recurrence before immunomodulation initiation highlights the dual role of neoplastic and immune mechanisms in PNP. Further research into tumor‐specific antigenic triggers and biomarker‐guided treatment strategies may refine management approaches for this complex disorder.

## Author Contributions

**Yongfeng Li:** conceptualization, data curation, writing – original draft. **Haochuan Ma:** funding acquisition, methodology, writing – original draft. **Xinsheng Chen:** conceptualization, data curation, formal analysis. **Yihong Liu:** investigation, software, supervision, validation. **Yu Zhang:** formal analysis, writing – original draft. **Junzhe Li:** writing – original draft. **Hanliang Zhang:** writing – original draft. **Liting Zhang:** writing – original draft. **Shuyun Xiong:** data curation, visualization, writing – original draft. **Jicai Chen:** conceptualization, data curation, funding acquisition, supervision, writing – review and editing.

## Ethics Statement

The authors have nothing to report.

## Consent

Written informed consent was obtained from the patient for publication of this case report and any accompanying images. A copy of the written consent is available for review by the Editor‐in‐Chief of this journal.

## Conflicts of Interest

The authors declare no conflicts of interest.

## Data Availability

Data sharing not applicable to this article as no datasets were generated or analysed during the current study.
